# 239. Sex Differences in Prosthetic Joint Infection

**DOI:** 10.1093/ofid/ofab466.441

**Published:** 2021-12-04

**Authors:** Christine M Mironenko, Milan Kapadia, Laura Donlin, Mark Figgie, Alberto V Carli, Michael Henry, Susan M Goodman, Andy O Miller

**Affiliations:** 1 Hospital for Special Surgery, New York City, New York; 2 Weill Cornell Medicine, New York, NY

## Abstract

**Background:**

Male sex has been demonstrated to be a non-modifiable risk factor for prosthetic joint infection (PJI) incidence in multiple studies. Given the known anatomical, genetic, and immunological differences between sexes, we compared the clinical characteristics of PJI among men and women.

**Methods:**

A retrospective cohort of total hip and knee arthroplasty PJIs from 2009 to 2019 were identified using a single institution PJI database. Included cases met the 2013 MSIS criteria. Microbiology, acuity (defined by implant age and symptom days), and surgical outcomes were collected. Success was defined as no further PJI surgery at two years. Continuous variables were tested with either Student’s t test or Mann-Whitney U test. Categorical variables were tested with either Chi-squared test or Fisher’s exact test.

**Results:**

We identified 1052 PJI patients, of whom 463 (44.0%) were women. In univariate analysis of the total cohort, women were younger (68.1 ± 11.2 vs 66.1 ± 11.8 years, p=0.01), had higher BMI (30.8 ± 7.78 vs 29.8 ± 6.0, p=0.04), and had a higher culture-negative rate (14.5% vs 9.0%, p < 0.01) than men, but no difference was noted in Charlson Comorbidity Index (Table 1). Among hip PJIs, women were likelier than men to present with acute PJI (15.9% vs 8.7%, p=0.03). There were no differences in debridement, antibiotics, and implant retention (DAIR) utilization (48.2% vs 44.1%, p=0.067), and overall treatment success (72.1% vs 71.6%, p=0.9), nor in any subanalysis of acute, hip, or knee PJIs.

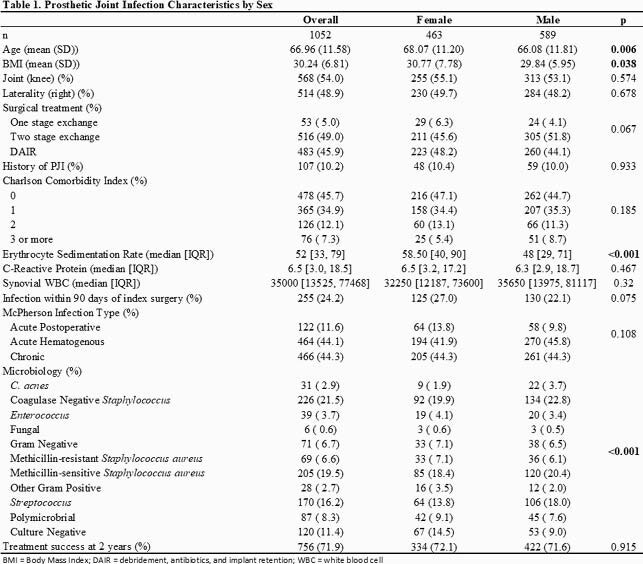

**Conclusion:**

Although females may present differently when diagnosed with PJI, overall outcomes and outcomes with respect to acuity and type of septic revision did not clearly differ in this single-center cohort. Further research in larger cohorts, including additional biomarkers and socioeconomic variables, may further elucidate relationships between sex and PJI characteristics including culture-negativity and symptom acuity.

**Disclosures:**

**All Authors**: No reported disclosures

